# Temporary trigeminal ganglion stimulation can improve zoster-related trigeminal neuralgia: a retrospective study in a single center

**DOI:** 10.3389/fneur.2024.1513867

**Published:** 2025-01-07

**Authors:** Kan Yue, Shengrong Xu, Xin Hu, Junhong Li, Ruilin He, Zongbin Jiang

**Affiliations:** Department of Pain Management, The Second Affiliated Hospital of Guangxi Medical University, Nanning, China

**Keywords:** trigeminal ganglion stimulation, zoster-related trigeminal neuralgia, trigeminal postherpetic neuralgia, varicella-zoster virus, neuromodulation

## Abstract

**Introduction:**

Conventional management approaches have been challenged in dealing with zoster-related trigeminal neuralgia. Percutaneous trigeminal ganglion stimulation (TGS) has been rarely reported as a potential treatment option for alleviating pain associated with this condition. The present study investigated the application of percutaneous TGS in a series of patients suffering from Zoster-related trigeminal neuralgia to evaluate its potential efficacy of pain relief.

**Methods:**

We retrospectively reviewed the medical records of all patients who underwent TGS at the Department of Pain Management, Second Affiliated Hospital of Guangxi Medical University. All patients were followed for up to 6 months. Clinical data, including the Visual Analog Scale (VAS), Pittsburgh Sleep Quality Index (PSQI), and medication consumption were recorded before and after treatment. Adverse events related to the treatment were also documented.

**Results:**

A total of nine patients underwent percutaneous TGS for Zoster-related trigeminal neuralgia. Among these patients, five (56%) experienced more than 50% pain relief at discharge. At the six-month follow-up, the mean VAS score decreased from preoperative 6.1 ± 1.5 to 2.5 ± 1.9, demonstrating a statistically significant reduction (*t* = 4.36, *p* < 0.05). The PSQI also showed a significant reduction from a baseline score of 14.1 to 6.5 at the six-month follow-up (*Z* = 4.2, *p* < 0.05). Seven patients reported satisfaction with the treatment and no serious adverse events occurred.

**Discussion:**

The results of the present study suggest that this contributes growing evidence that percutaneous TGS may be an effective treatment for Zoster - related trigeminal neuralgia.

## 1 Introduction

Trigeminal herpes zoster is typically characterized as a painful vesicular rash that follows the distribution of the trigeminal nerve. This condition arises from the reactivation of the latent varicella-zoster virus within the trigeminal ganglion. It is estimated that approximately 1 million new cases occur annually in the United States, with up to 20% involving branches of the trigeminal nerve (1). Symptoms of trigeminal herpes zoster include itching, tingling, or pain sensation in the rash, burning and tingling sensations on the affected side of the face (paresthesia/dysesthesia), sharp, shooting pain in response to light touch (allodynia), and prolonged or exaggerated pain responses (hyperalgesia/hyperesthesia) (2). The most common complication of herpes zoster is postherpetic neuralgia (PHN). Notably, herpes zoster occurring in the ophthalmic distribution is associated with a higher risk of PHN compared to other distributions of herpes zoster (3). Trigeminal postherpetic neuralgia (TG-PHN) is defined as facial pain caused by herpes zoster that persists or recurs for at least 3 months after the initial infection, affecting one or more branches of the trigeminal nerve. Zoster-related trigeminal neuralgia in the facial region is often more intense than pain experienced in other areas of the body, primarily due to the high concentration of nerve endings in the facial region. Additionally, the visibility of herpes zoster outbreaks on the face can lead to considerable psychological distress, which further complicates the management of facial herpetic neuralgia (4). Given the impact on the orofacial region, zoster-related trigeminal neuralgia can lead to significant functional and social challenges, including difficulties with eating and speaking. Therefore, effective management of this condition is essential.

The initial treatment for zoster-related trigeminal neuralgia typically involves a range of medications, including antiviral drugs, anticonvulsants, nonsteroidal anti-inflammatory drugs (NSAIDs), tricyclic antidepressants, and opioid analgesics. However, the use of oral medications may result in adverse effects, such as nausea, vomiting, and dizziness (5, 6). It is important to note that antiviral medications do not prevent PHN (7). Furthermore, some individuals experiencing severe pain may not respond adequately to oral medications (8). According to previous studies, the initial success rates for these medications are estimated to be about 50% (9). In cases where patients do not respond to pharmacological therapy, interventional treatments—such as nerve block and neurodestructive procedures targeting the trigeminal nerve—are considered (6, 10). Studies have shown that nerve blocks can provide a 50% pain reduction in 66.7% of patients with zoster-related trigeminal neuralgia (11). Nevertheless, nerve blocks only provide a relatively shorter duration of pain relief. In addition, a meta-analysis showed that there was no significant difference in pain relief rates between the pulsed radiofrequency group and the control group in patients with zoster-related trigeminal neuralgia (12). Various ablative neurosurgical procedures have been applied in trigeminal neuropathy. Previous studies have indicated that neuro-destructive interventions had little beneficial effect and exacerbated pain symptoms in 73% of patients (13). Peripheral nerve stimulation has been used in the management of zoster-related trigeminal neuralgia, providing at least 50% pain relief in 70–80% of patients (8). Given that neuro-destructive techniques may exacerbate pain, functional neuromodulation techniques may be the most effective treatment option for patients suffering from zoster-related trigeminal neuralgia. High cervical spinal cord stimulation (SCS) has been used to treat headache and facial pain, nevertheless, strong evidence supporting its efficacy is lacking (14). Various studies have shown that multiple revision surgeries are required after SCS implantation (15). In 2022, the Neurostimulation Appropriateness Consensus Committee did not recommend the use of SCS for trigeminal neuralgia (16). The trigeminal ganglion is anatomically well targeted, TGS has proven to be a safer and more reliable option. It has the advantage of being less invasive and easier to perform the procedure.

The trigeminal nerve is responsible for transmitting sensory signals from most areas of the face, mouth, nose, meninges, and facial muscles, as well as for delivering motor commands to the masticatory muscles (17). This nerve has three branches: the ophthalmic, maxillary, and mandibular branches, which convey sensory information to the trigeminal ganglion. The trigeminal ganglion predominantly consists of the pseudounipolar cell bodies of the exteroceptors within the somatosensory system and serves as a significant target for neuromodulation. The analgesic mechanism may be based on the “gate control theory,” which suggests that non-painful stimulation of the first relay site for craniofacial afferent nociceptors of the peripheral trigeminal branches can inhibit the transmission of pain signals to the central nervous system (4). TGS has proven particularly effective in treating patients with chronic trigeminal neuropathic pain and persistent idiopathic facial pain who have either failed conventional surgical interventions or have been deemed unsuitable candidates for such procedures (18). For patients with trigeminal neuralgia and refractory facial pain, permanent electrical stimulation implants are administered following successful testing. Current evidence supports this approach as a promising therapeutic modality for managing trigeminal neuralgia. However, there are currently limited clinical reports regarding the efficacy of TGS for zoster-related trigeminal neuralgia. Additionally, the implantation of permanent electrodes imposes a significant financial burden on patients, and there are esthetic concerns associated with this procedure. Furthermore, considering that the analgesia produced by SCS often extends beyond the stimulation period (19) and that PHN resolves within a year in most patients (78%) (3), it is important to investigate that whether temporary TGS would be effective in alleviating pain associated with trigeminal herpes zoster. Our team conducted a retrospective analysis of patients who received TGS treatment at our center. The primary objective of this study was to evaluate the effectiveness of temporary TGS in managing patients with zoster-related trigeminal neuralgia. This research aims to provide evidence supporting the use of temporary TGS for this pain condition.

## 2 Materials and methods

### 2.1 Ethical approval of the study protocol

The study protocol was approved by the human ethics committee of the Second Affiliated Hospital of Guangxi Medical University.

### 2.2 Methods

A retrospective analysis was conducted on the clinical data of patients with zoster-related trigeminal neuralgia who underwent TGS treatment at the Department of Pain Management of the Second Affiliated Hospital between 2020 and 2024. The baseline characteristics of these patients were recorded, including the site of pain, pain intensity, duration, prior pain management strategies, quality of sleep and medication intake. Prior to undergoing TGS, each patient underwent a neuropsychiatric evaluation to rule out other psychiatric causes. We contacted each patient by telephone to gather the data on pain intensity, sleep quality, and medication intake at follow-up time points after the implantation of the TGS neuromodulation system. Furthermore, any adverse events were documented during the follow-up period.

### 2.3 Surgical technique

**Figure 1:**
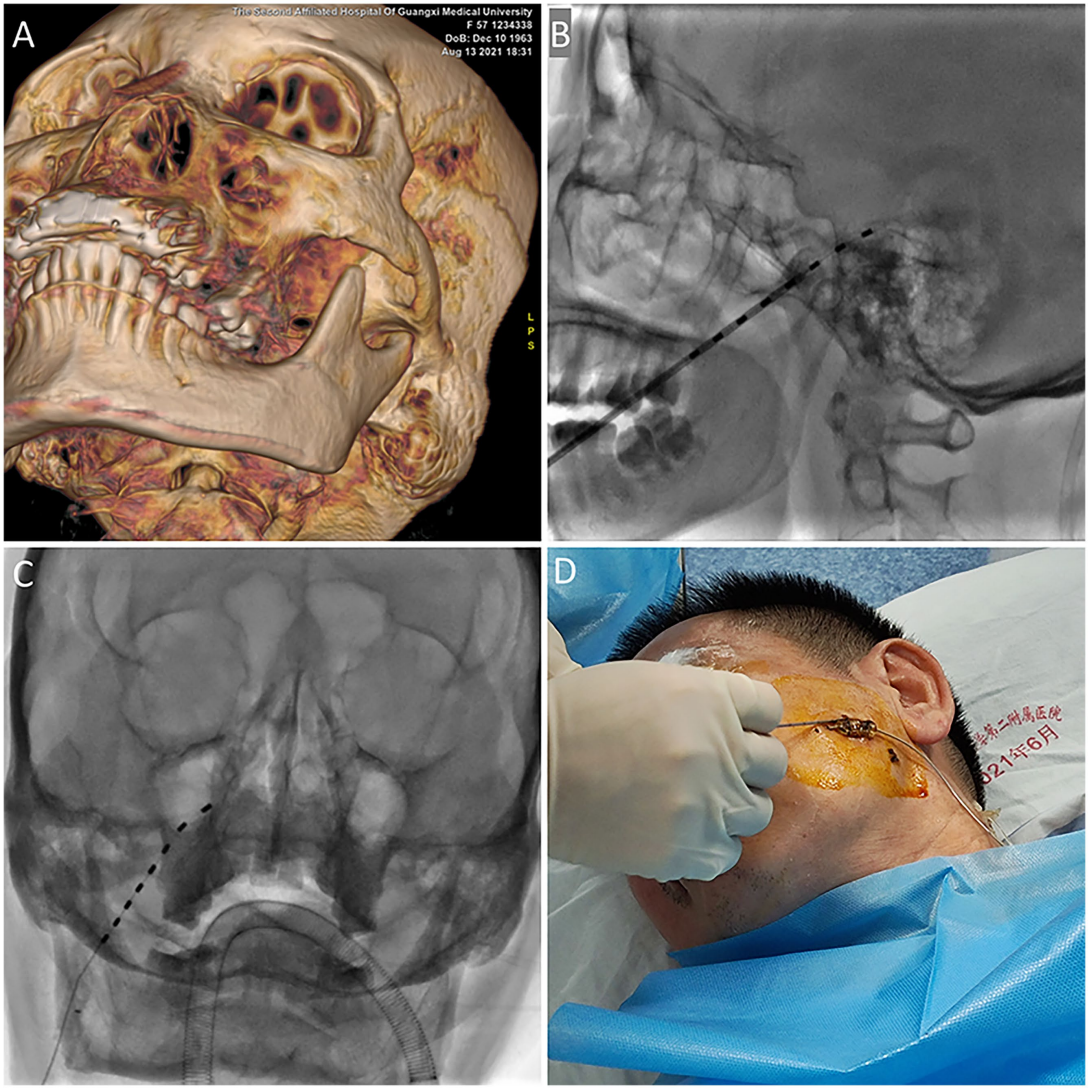
**(A)** The foramen ovale was identified through three-dimensional reconstruction. **(B)** The electrode was advanced until the tip of the most distal contact reached the clivus. **(C)** An anteroposterior radiographic image of the percutaneous TGS with lead placement is presented. **(D)** The electrode was anchored using a soft silastic anchor. TGS: trigeminal ganglion stimulation.

The technique of insertion was identical to that used for percutaneous radiofrequency ablation of trigeminal neuralgia. Given the minimally invasive nature of the procedure and the necessity for intraoperative testing, we favor the use of local anesthesia. However, some patients may require general anesthesia, and in such cases, we conduct the test while the patient is awake. The patient was positioned supine on the operating table, with the head slightly extended. The foramen ovale was visualized fluoroscopically using three-dimensional reconstruction and the puncture site was determined (Figure 1A). Following the administration of local anesthesia, an 18G needle was inserted approximately 2.5 cm lateral to the labial commissure, aiming toward the foramen. Upon entering the foramen, the stylet was removed, and the electrode (Medtronic model no. 977D60) was introduced until the tip of the most distal contact reached the clivus (Figures 1B,C). Intraoperative test stimulation was performed, and the electrode position was adjusted until stimulation paresthesias were perceived in the usual area of pain. The needle was then withdrawn under fluoroscopic control to ensure the maintenance of the electrode position. Subsequently, the electrode was anchored to the subcutaneous tissue and fascia using a soft silastic anchor, and it was connected to an external pulse generator (EPG) (Figure 1D). The most distal contact (0) is programmed to be negative, while the second contact (1) is configured to be positive. Subsequently, the contact configuration is adjusted to optimize coverage of the patient’s painful area. The initial amplitude during programming is typically set to a low value, and similar considerations apply to the programming of pulse width as they do to amplitude (20). Electrical stimulation parameters were tailored to the patient’s pain condition, with a frequency ranging from 40 to 60 Hz, pulse width ranging from 60 to 540 μs, and voltage set between 0.2 and 0.9 V. To reduce the risk of infection, the duration of stimulation was set at 14 days, as recommended by the consensus of Chinese experts (21).

### 2.4 Evaluation and outcomes

Follow-up evaluations were performed at 1, 3, and 6 months post-treatment. Patients rated their average pain intensity in the past 7 days using VAS ranging from 0 to 10, where 0 indicates no pain and 10 represents the worst pain imaginable. Sleep quality was assessed using the Pittsburgh Sleep Quality Index (PSQI), a tool designed to evaluate sleep quality over a one-month period. The PSQI consists of 19 questions that assess seven distinct components: subjective sleep quality, sleep latency, sleep duration, habitual sleep efficiency, sleep disturbances, use of sleep medication, and daytime dysfunction. The scores for these components were summed to calculate the total PSQI score, which ranges from 0 to 21, with higher scores signifying poorer sleep quality. The dosage of pregabalin administered to patients was documented both preoperatively and at the last follow-up visit. Safety was assessed by identifying adverse events associated with the device and/or the lead implant procedure. Additionally, patients were asked whether the implantation of a stimulation device in the facial area had any impact on their lives and whether they experienced masseter contractions. Patients were also asked to what extent they were satisfied with the treatment and whether they would choose the same treatment again for similar outcomes. Based on their response (yes, no or not sure), patients were categorized as satisfied, dissatisfied or uncertain.

### 2.5 Statistical analysis

Continuous variables were described as mean ± standard deviation, and were initially assessed for normality using the Shapiro–Wilk test. Data in line with a normal distribution were compared between pre- and post-treatment using ANOVA, followed by *post hoc* Bonferroni test. Data exhibiting a skewed distribution were analyzed using Wilcoxon signed-rank tests for comparisons between pre- and post-treatment. The pregabalin dose at baseline and 6-month follow-up were assessed by two-tailed student’s *t*-test. The significance level was set at 0.05. All analyzes were conducted using Prism Version 9.

## 3 Results

### 3.1 Characteristics of patients at baseline

The study did not reach the calculated sample size and we conducted a preliminary exploratory analysis of the data. This study included all nine patients who underwent the implantation of TGS devices. All the patients completed the 14-day stimulation period. The baseline characteristics are presented in Table 1. The mean (SD) age of the patients was 68.6 (9.9) years, with four patients being female. Three patients had comorbidities such as type 2 diabetes, while one patient had a history of lung cancer surgery. All patients presented with pain associated with the trigeminal nerve following an initial herpetic skin rash, characterized by burning, throbbing, stabbing, and shocking pain. Prior to the procedure, patients had failed various treatments, including analgesics, anticonvulsants, tricyclic antidepressants, sympathetic nerve blocks (SGB) and radiofrequency. The mean duration of pain symptoms experienced by the patients before TGS implantation was 169 days. The trigeminal nerve pain was distributed as follows: three patients experienced pain in the third division, two patients in all three divisions, two patients in the first and second divisions, and two patients in the second and third divisions. At baseline, the average VAS score was 6.1 ± 1.5.

**Table 1:** Demographic and clinical characteristics of patients.

Characteristic	Value
Age (years), mean (SD)	68.6 (9.9)
Range	57–86
**Gender, *n* (%)**	
Male	5 (56%)
Female	4 (44%)
**Pain duration (days)**	
Mean ± SD	169 ± 215
Median (range)	60 (14–730)
**Location, *n* (%)**	
V1 and V2	2 (22%)
V2 and V3	2 (22%)
V3	3 (33%)
V1, V2, and V3	2 (22%)
Pain scores at baseline, VAS, mean (SD)	6.1 (1.3)

SD, standard deviation; V1, ophthalmic nerve; V2, maxillary nerve; V3, mandibular nerves.

### 3.2 Pain severity

**Figure 2:**
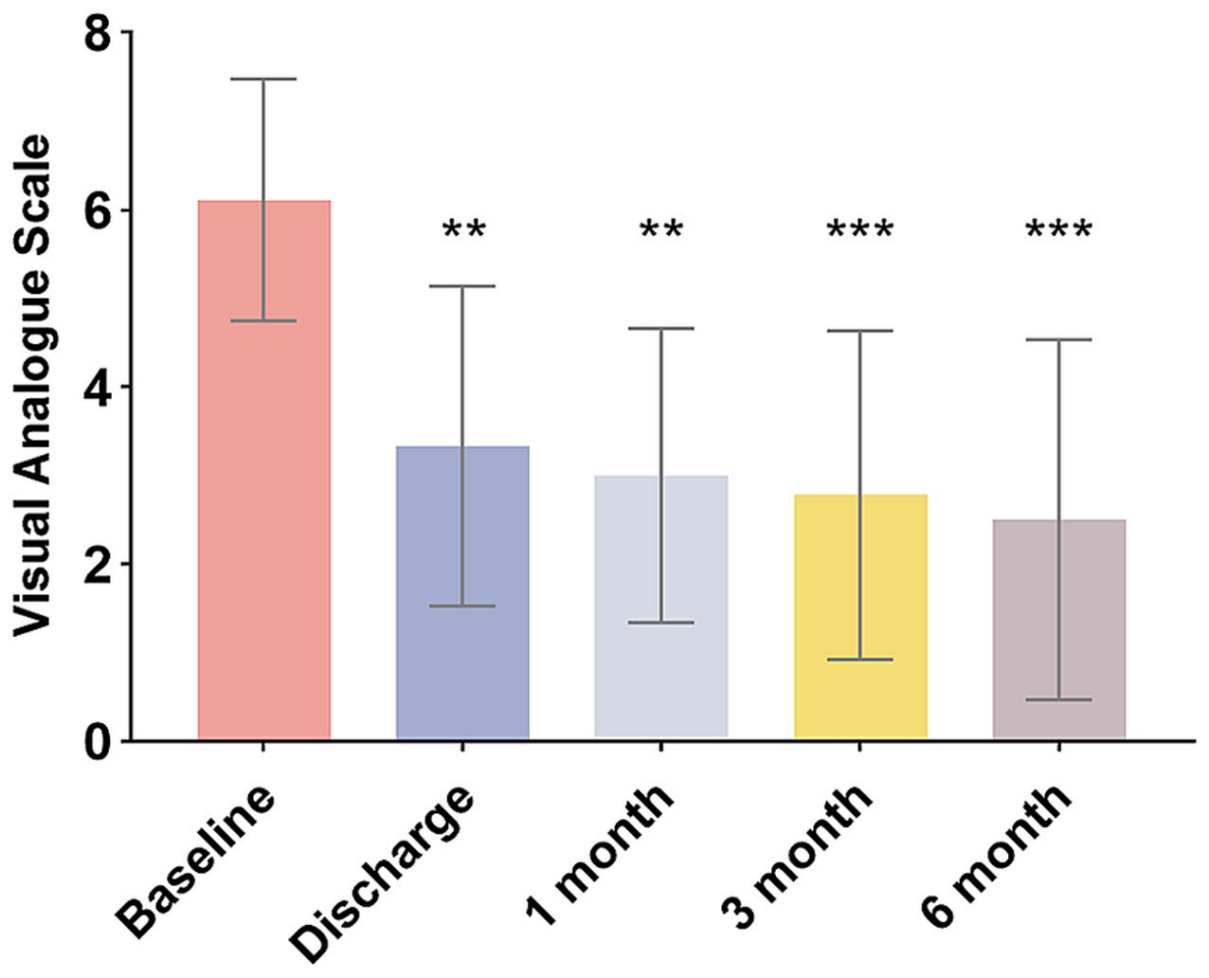
Changes in VAS scores of patients over different periods. **p < 0.01, ***p < 0.001.

A significant reduction in VAS scores was observed at all postoperative periods compared to the baseline (*F* = 6.275, *p <* 0.05), indicating a noteworthy decrease in pain intensity (Figure 2). At discharge, the average VAS was 3.3 ± 1.7. On average, the percentage of pain reduction was 44.9% (30.3%). Five patients achieved good pain relief outcomes (defined as pain relief greater than 50%), resulting in a response rate of 56% among the nine patients. In one case, complete pain relief was achieved. Three out of the nine patients exhibited poor outcomes, with pain relief recorded at less than 50%. In two instances, pain scores remained unchanged, leading both patients to subsequently undergo radiofrequency thermocoagulation. After a follow-up period of 6 months, the patients’ VAS scores decreased to an average of 2.5, with two patients reporting complete pain relief.

### 3.3 Sleep quality

**Figure 3:**
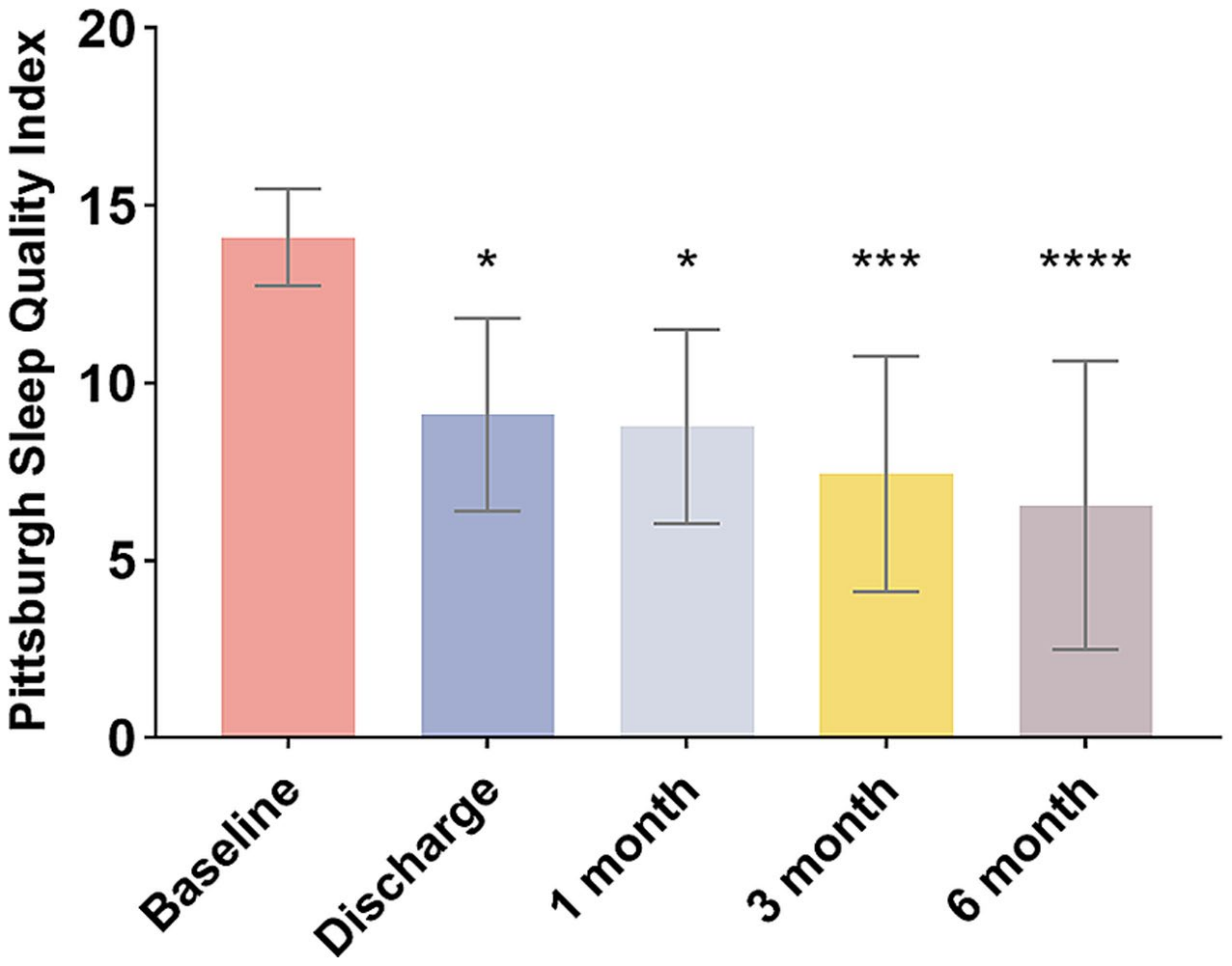
Changes in PSQI scores of patients over different periods. *p < 0.05, ***p < 0.001, ****p < 0.0001.

The patients’ PSQI score at baseline was reported as 14.1 ± 1.3. The postoperative PSQI scores were significantly lower than baseline values at each observation time point (*p* < 0.05). The mean PSQI score for nine patients was significantly reduced to 6.6 at the latest follow-up (Figure 3), indicating a substantial improvement in sleep quality.

### 3.4 Pregabalin dose

**Figure 4:**
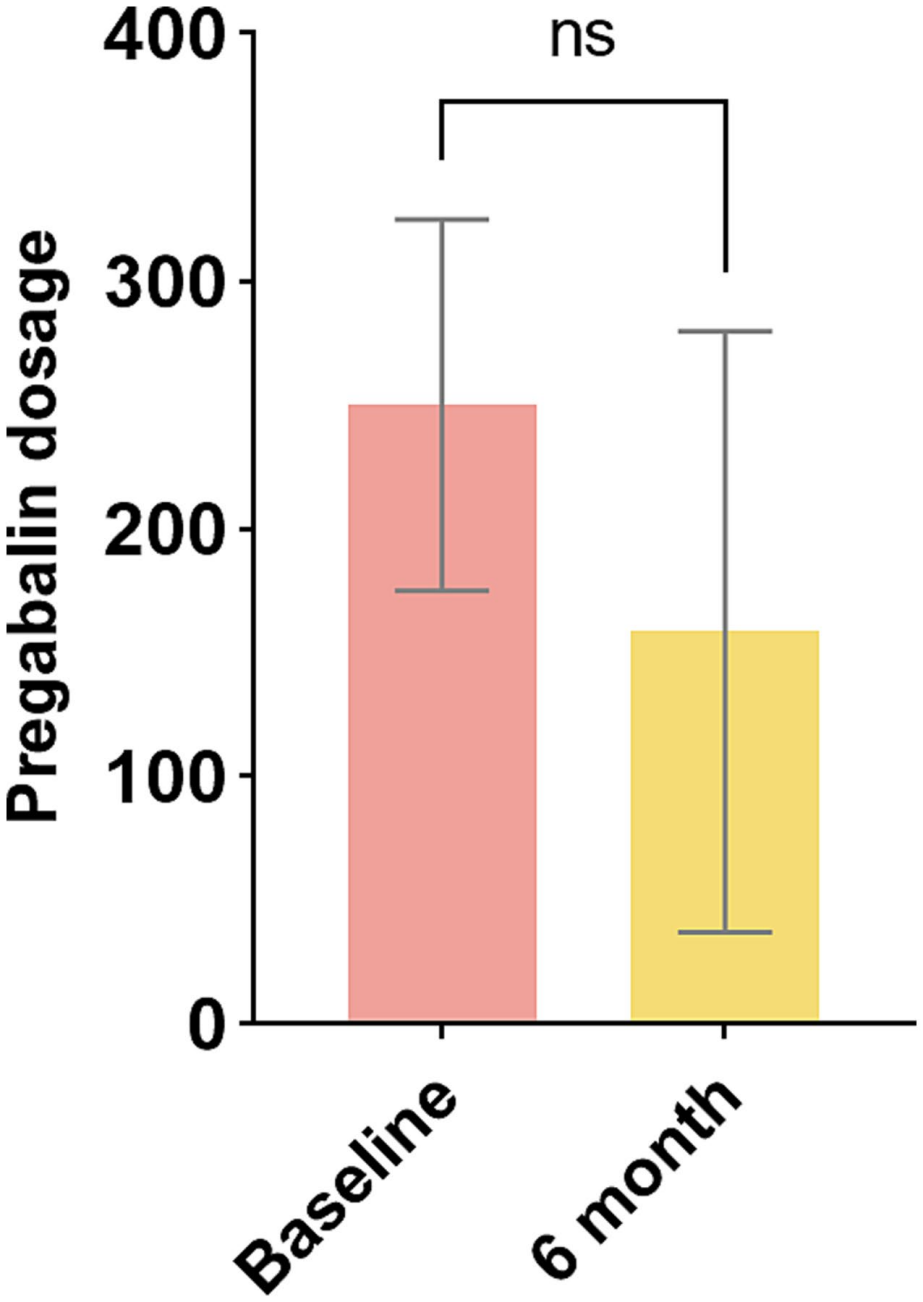
Changes in pregabalin dosage. ^ns^*p* > 0.05.

In this study, the dosages of pregabalin administered to patients were examined. Figure 4 compares the intake of pregabalin. The pregabalin doses at baseline and at six-month follow-up were 250.0 mg and 158.3 mg, respectively. There was no significant variance between the baseline and the final follow-up (*p* > 0.05). Two patients no longer required medication, while three patients had their daily dosage requirements reduced.

### 3.5 Adverse events

We did not observe any serious complications, including cerebrospinal fluid leakage, intracranial infection, or intracranial hemorrhage. Furthermore, there were no complications related to the hardware devices, such as electrode displacement.

### 3.6 Patients satisfaction

No patients experienced an exacerbation of pain due to the stimulation, and none reported contractions of the masseter muscle. Additionally, no patients indicated any adverse effects of the electrical stimulation devices on eating, speaking, or related activities. Among the nine patients, seven expressed satisfaction with the TGS treatment, while two patients were unsure.

## 4 Discussion

In the present study, 56% of patients reported more than 50% pain relief following TGS. At the six-month follow-up, the VAS scores indicated a statistically significant decrease of approximately 3.6 points, while the PSQI showed a significant reduction compared to baseline levels. Although a statistically significant difference was not achieved, we observed a decrease in pregabalin consumption compared to preoperative levels. No obvious adverse effects, such as infection, hemorrhage, or lead migration were observed. These findings highlight the potential feasibility of percutaneous TGS as a treatment approach for zoster-related trigeminal neuralgia. The current study suggests that temporary TGS is both effective and safe for the management of zoster-related trigeminal neuralgia.

TGS has been reported to be effective in alleviating facial pain. However, there are limited studies addressing its efficacy in patients suffering from herpes zoster-related pain within the trigeminal nerve distribution area, and the findings are inconsistent. Taub et al. (22) studied the effect of TGS on 34 patients with chronic refractory facial pain, including 4 patients with PHN. The results indicated that pain relief in these 4 patients did not reach 50%. Mehrkens’ long-term follow-up of patients with refractory trigeminal neuropathy treated with TGS revealed that only 33% of patients with PHN reported satisfaction with their treatment. Notably, 67% of these patients experienced a worsening of their condition (23). Recently, Chang et al. (24) presented a retrospective series of patients who underwent short-term TGS for VZV-related pain, revealing a significant decrease in VAS scores after treatment. Niu et al. (8) reported a case of postherpetic trigeminal neuralgia in which the patient’s VAS decreased from 9 to 1 after short-term TGS. Gupta also documented a case of postherpetic trigeminal neuralgia, noting a 50% reduction in pain following TGS (25). The published reports are retrospective reviews or case reports performed to assess the efficacy and safety of TGS in the treatment of PHN. Currently, to the best of our knowledge, there are no randomized controlled clinical trials to investigate the effect of TGS on zoster-related trigeminal neuralgia.

The rates of successful stimulation at 2 weeks reported in our study were 56%. Xu et al. (26) reported response rates of 33.3, 66.7, and 83.3% at 1 week, 4 weeks, and 8 weeks, respectively. These findings suggest that the pain relief effects of TGS tend to increase over time. Our response rate is consistent with the findings of Xu et al., but it is lower than those previously published data in facial pain, which ranges between 66 and 77.4% (27). Possible explanations for this discrepancy include the small sample size utilized in our study. Additionally, herpes zoster-induced pain may have a slightly poorer prognosis compared to iatrogenic and traumatic neuropathic pain.

Our results indicated that the VAS decreased by an average of 3.6 points from baseline to the 6-month follow-up. These findings are consistent with those of Chang et al. (24) who reported a decrease from 7.41 at baseline to 4.41 at the 12-week follow-up. Xu et al. (26) utilized short-term TGS to manage herpetic neuralgia within the trigeminal nerve distribution area and showed a mean reduction in pain scores of 5.8 at the 24-week follow-up compared to preoperative measurements. The greater reduction in pain scores observed in Xu et al.’s study may be attributed to their inclusion of only patients with second and third branch pain, whereas our study also included patients with pain in the first branch distribution area. Managing pain associated with ophthalmic herpes zoster is challenging. It is notable that the incidence of PHN is higher in herpes zoster ophthalmicus (HZO) than in other forms of herpes zoster (7). Furthermore, some patients in Xu et al.’s study received 1,000 Hz high-frequency stimulation. Previous research suggests that high-frequency stimulation may yield superior analgesic effects compared to the traditional TONIC mode (28). This aspect warrants further investigation in future studies.

Among the nine patients included in this study, two did not exhibit significant changes in their postoperative pain scores. One of these patients was a 58-year-old female suffering from zoster-related pain affecting all three branches of the right trigeminal nerve, while the other was a 66-year-old male with a history of pain lasting over 2 years. Zoster involving all three branches of the trigeminal nerve suggests that the patient may have underlying immune dysfunction (29). The immune system plays a critical role in pain regulation through the release of molecular mediators (30). We speculate that the primary reason for Patient 1’s poor response to TGS is neuronal sensitization resulting from immune dysfunction. Furthermore, the involvement of multiple branches has resulted in significant nerve damage, making it challenging to reverse the damage through TGS (31). Research indicates that patients with a longer duration of PHN often experience a poorer prognosis (32). Chronic pain can induce central sensitization of the brain and altered central plasticity (33). The prolonged duration of pain in Patient 2 resulted in central sensitization, which may have contributed to the poor outcome.

Results from the current study indicate that patients experienced a continued improvement in sleep quality with 7.5 point reduction in PSQI scores. Consistent with the findings of Xu et al. (26), there was a significant reduction in PSQI scores compared to baseline. The improvement in sleep quality following TGS treatment may be attributed not only to pain relief but also to the influence of TGS on the structure and function of the central nervous system. Results from Bu et al. indicated that one of the mechanisms underlying the improvement in sleep may be related to changes in the orbitofrontal cortex (34). Furthermore, the research conducted by De Groote et al. (35) demonstrated that the increased strength of functional connectivity between the left dorsolateral prefrontal cortex and the right anterior insula was significantly correlated with the minimum clinically important difference (MCID) value of the PSQI following spinal cord stimulation (SCS) treatment. Additionally, spinal electrical stimulation may modulate the medial pain signaling pathway and positively affect the emotional dimensions associated with pain (36). The study by Niu et al. showed that, following TGS treatment, patients not only reported a decrease in pain scores but also exhibited significant improvements in anxiety and depression. Moreover, spinal cord electrical stimulation has the potential to alter the functional connectivity between various brain regions (35). While these studies are outside the trigeminal ganglion, it is plausible that the trigeminal ganglion probably serves a similar role in pain transmission from the trigeminal branches as the dorsal root ganglia do for the body. Consequently, TGS may exert effects on the central nervous system analogous to those of spinal cord stimulation.

The trigeminal ganglion emerged as a target for electrical stimulation in the treatment of trigeminal neuralgia (TN) in the late 1970s. Initially described by Steude (37), this modality has evolved into a valuable treatment option for various types of facial pain. This approach is advantageous due to its minimally invasive nature and its ability to provide focal coverage in the territory of a single peripheral branch of the trigeminal nerve. However, the mechanism of pain relief associated with TGS remains unclear. Spinal cord stimulation is based on the gate control theory, which posits that pain control can be achieved by selectively activating large, rapidly conducting fibers (38). While the pain associated with the trigeminal system has not been studied as extensively as spinal cord mediated pain, it is believed that the mechanisms of pain modulation and conduction pathways are quite similar (39). The trigeminal ganglion contains the cell bodies of sensory neurons that convey information about touch, pain and temperature in the face and axons of sensory neurons that convey information about proprioception traveling through the ganglion toward their cell bodies in the brain-stem (39). Consequently, appropriate stimulation of these A-fiber nerves may inhibit or diminish the transmission of pain signals.

The analgesic action may also be related to the normalization of sensation. Lazorthes et al. (40) evaluated changes in the sensory function of the trigeminal nerve with TGS, finding that it can normalize or improve pain sensation following short period of continuous stimulation. Electrophysiological studies indicate that pain-evoked potentials resulting from painful stimuli can be completely suppressed by TGS of the corresponding trigeminal division (23). Furthermore, positron emission tomography (PET) studies demonstrate that TGS significantly affects pain modulation pathways (41). A severe inflammatory reaction caused by replication of the varicella-zoster virus latent within the trigeminal ganglion can lead to neuropathic pain (8). Accumulating evidence suggests that neuromodulation can regulates inflammation and neuroinflammation in trigeminal ganglia through neuro-immune interactions (42). In summary, the mechanism through which TGS exerts its analgesic effect remains unclear, highlighting the need for further in-depth research in the future.

The use of effective programming strategies is critical to the success of neurostimulation surgical treatment. Currently, essential details regarding in programming strategies are often inadequately described. Different authors provide varying recommendations for setting stimulation parameters. Higher amplitudes tend to activate the masseter muscle, resulting in contractions of the jaw, which can cause discomfort for the patient (43). So Buyten recommends that the most comfortable stimulation parameters are a frequency of 50 Hz, a pulse width of 450 μs, and an amplitude ranging from 0.2 to 2.5 V (13). Gupta et al. (44) discovered that patients with trigeminal neuralgia were unable to tolerate lower frequency and higher pulse width stimulation parameters (40–80 Hz, 120–200 us), leading them to advocate for the kilohertz stimulation mode. Furthermore, they observed that patients experienced difficulty tolerating increased voltage, with a recommended voltage range of 0.5–3 volts. Machado et al. (45) reported that most patients generally preferred amplitudes near 1 V. In this study, the amplitude set for patients did not exceed 1 V. Therefore, the patients did not experience significant paraesthesia and were satisfied with the TGS treatment. Additionally, the location of the trigeminal ganglion within the bony structure of Meckel’s cave necessitates that electrical stimulation be precisely targeted. Utilizing high-frequency with lower pulse width and voltages can limit the spatial extent of stimulation, thereby concentrating the effects on the intended target while minimizing the impact on surrounding tissue (24). However, the ideal parameters for the treatment of zoster-related trigeminal neuralgia remain to be verified.

Percutaneous TGS is generally regarded as a safe surgical procedure, and with the overall rate of surgical complications in TGS being remarkably low. The most common complication associated with TGS is electrode dislocation, which can be attributed to the unique anatomy of the foramen ovale. Studies indicate that the incidence of electrode dislocation is approximately 10%, and this frequency is correlated with the diameter of the electrode (23). Larger electrodes appear to be less prone to displacement, however, they are associated with a higher likelihood of eliciting dysesthesia (13). In our case series, we did not observe any instances of electrode migration. This may be attributed to the short duration of implantation and the immobilization measures that were implemented.

Peripheral nerve stimulation has also been widely used for trigeminal neuropathic pain, however, it has had little success in treating pain in the V3 distribution (46). Moreover, electrode fractures are prone to occur due to the movement of the mandible (47). Our findings suggest that TGS is effective for managing pain in the V3 distribution.

## 5 Limitation

Our findings must be interpreted with caution due to the limitations of this study. Firstly, this study is a single-center retrospective analysis with a limited sample size, which may impact the generalizability of our conclusions. Secondly, the inherent limitations of a retrospective case series necessitate careful interpretation of our findings. Additionally, the follow-up period was not sufficiently lengthy. There is a continued need for large-scale, randomized, placebo-controlled clinical trials to further evaluate the efficacy and safety of the treatments.

## 6 Conclusion

TGS is a promising therapeutic alternative for patients suffering from pain. However, Future research should concentrate on optimizing neuromodulation parameters, particularly concerning the safety and efficacy of high-frequency and burst stimulation in the management of Zoster-related trigeminal neuralgia.

## Data Availability

The original contributions presented in the study are included in the article/Supplementary material, further inquiries can be directed to the corresponding author.

## Supplementary material

Figure S1 Visual analog scale scores before and after TGS.

Figure S2 PSQI before and after TGS.
