# Supplementary figures and images for: Temporary trigeminal ganglion stimulation can improve zoster-related trigeminal neuralgia: a retrospective study in a single center
Source: Front Neurol. 2025 Jan 7;15:1513867. doi: 10.3389/fneur.2024.1513867 (PMC11747470; doi:10.3389/fneur.2024.1513867)

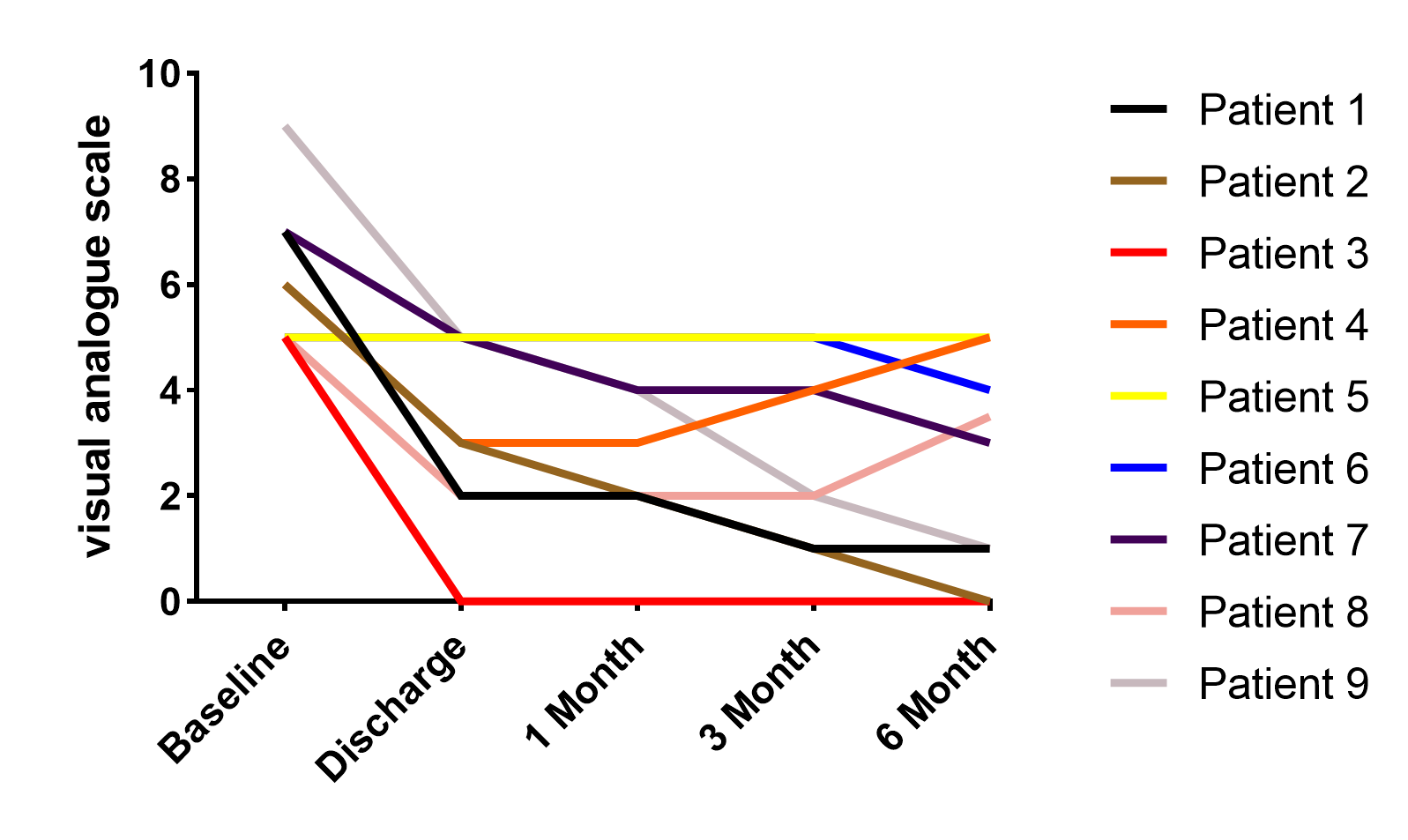

Supplement: Figure S1 — Visual analog scale scores before and after TGS. [file Image_1.TIF]

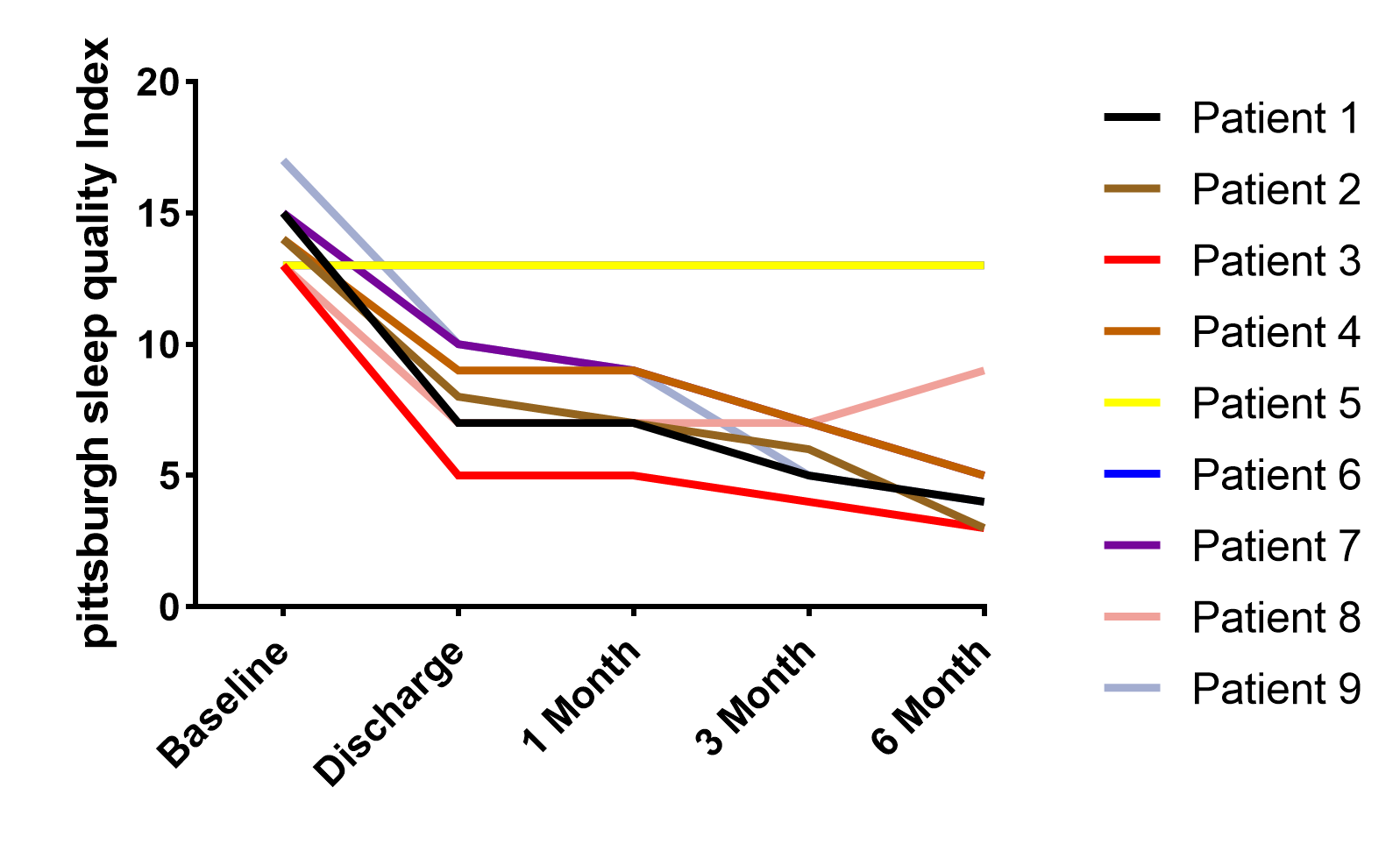

Supplement: Figure S2 — PSQI before and after TGS. [file Image_2.TIF]
