# Supplementary material for: Temporary trigeminal ganglion stimulation can improve zoster-related trigeminal neuralgia: a retrospective study in a single center
Source: Front Neurol. 2025 Jan 7;15:1513867. doi: 10.3389/fneur.2024.1513867 (PMC11747470; doi:10.3389/fneur.2024.1513867)
Supplement: Supplementary file 3 [file Table_1.DOCX]

**Table 1 Pre- and post-operative visual analogue scale (VAS) in patients undergoing TGS**

| Patient | Baseline | Discharge | 1 month | 3 month | 6 month |
| --- | --- | --- | --- | --- | --- |
| 1 | 7 | 2 | 2 | 1 | 1 |
| 2 | 6 | 3 | 2 | 1 | 0 |
| 3 | 5 | 0 | 0 | 0 | 0 |
| 4 | 6 | 3 | 3 | 4 | 5 |
| 5 | 5 | 5 | 5 | 5 | 5 |
| 6 | 5 | 5 | 5 | 5 | 4 |
| 7 | 7 | 5 | 4 | 4 | 3 |
| 8 | 5 | 2 | 2 | 2 | 3.5 |
| 9 | 9 | 5 | 4 | 2 | 1 |
