# Supplementary material for: Temporary trigeminal ganglion stimulation can improve zoster-related trigeminal neuralgia: a retrospective study in a single center
Source: Front Neurol. 2025 Jan 7;15:1513867. doi: 10.3389/fneur.2024.1513867 (PMC11747470; doi:10.3389/fneur.2024.1513867)
Supplement: Supplementary file 4 [file Table_2.DOCX]

**Table 2 Pre- and post-operative Pittsburgh Sleep Quality Index (PSQI) in patients undergoing TGS**

| Patient | Baseline | Discharge | 1 month | 3 month | 6 month |
| --- | --- | --- | --- | --- | --- |
| 1 | 15 | 7 | 7 | 5 | 4 |
| 2 | 14 | 8 | 7 | 6 | 3 |
| 3 | 13 | 5 | 5 | 4 | 3 |
| 4 | 14 | 9 | 9 | 7 | 5 |
| 5 | 13 | 13 | 13 | 13 | 13 |
| 6 | 13 | 13 | 13 | 13 | 13 |
| 7 | 15 | 10 | 9 | 7 | 5 |
| 8 | 13 | 7 | 7 | 7 | 9 |
| 9 | 17 | 10 | 9 | 5 | 4 |
